# Mucosal vaccination with a live recombinant rhinovirus followed by intradermal DNA administration elicits potent and protective HIV-specific immune responses

**DOI:** 10.1038/srep36658

**Published:** 2016-11-17

**Authors:** Khamis Tomusange, Danushka Wijesundara, Jason Gummow, Steve Wesselingh, Andreas Suhrbier, Eric J. Gowans, Branka Grubor-Bauk

**Affiliations:** 1 Virology Laboratory, Basil Hetzel Institute, Discipline of Surgery, University of Adelaide, Adelaide, South Australia, Australia; 2 South Australian Health and Medical Research Institute, Adelaide, South Australia, Australia; 3 QIMR Berghofer Medical Research Institute, Brisbane, Queensland, Australia

## Abstract

Mucosal immunity is deemed crucial to control sexual transmission of human immunodeficiency virus (HIV). Herein we report the efficacy of a mucosal HIV vaccine strategy comprising intranasal (IN) vaccination with a cocktail of live recombinant human rhinoviruses (HRVs) encoding overlapping fragments of HIV Gag and full length Tat (rHRV-Gag/Tat) followed by intradermal (ID) vaccination with DNA vaccines encoding HIV Gag and Tat (pVAX-Gag-Tat). This heterologous prime-boost strategy will be referred to hereafter as rHRV-DNA. As a control, IN vaccination with wild type (wt)-HRV-A1 followed by a single ID dose of pVAX (wt-HRV-A1/pVAX vaccination) was included. rHRV-DNA vaccination elicited superior multi-functional CD8^+^T cell responses in lymphocytes harvested from mesenteric lymph nodes and spleens, and higher titres of Tat-specific antibodies in blood and vaginal lavages, and reduced the viral load more effectively after challenge with EcoHIV, a murine HIV challenge model, in peritoneal macrophages, splenocytes and blood compared compared with wt-HRV-A1/pVAX vaccination or administration of 3 ID doses of pVAX-Gag-Tat (3X pVAX-Gag-Tat vaccination). These data provide the first evidence that a rHRV-DNA vaccination regimen can induce HIV-specific immune responses in the gut, vaginal mucosa and systemically, and supports further testing of this regimen in the development of an effective mucosally-targeted HIV-1 vaccine.

A potential reason why previous HIV vaccine trials were ineffective^1^,^2^,^3^ or modestly effective^4^ is the failure to generate effective mucosal immunity^5^. A majority of HIV transmissions occur via genito-urinary or genito-rectal mucosal surfaces^6^ and extensive CD4^+^ T cell depletion occurs in the gastrointestinal mucosa after infection^7^. This highlights the need to develop vaccines capable of eliciting protective HIV-specific immune responses at these surfaces. Moreover, previous HIV vaccine strategies focused on systemic vaccination, which induced little or no protective immune responses at the mucosa^5^. Therefore, mucosal vaccination strategies able to elicit HIV-specific immunity both systemically and at mucosal surfaces are being actively pursued^8^.

Ideally, mucosal HIV vaccines should generate broadly neutralizing antibodies (bNAbs) against the envelope proteins to prevent primary HIV infection as has been observed in animal studies involving passive transfer of broadly neutralizing antibodies^9^. However, due to the recognised difficulties in designing vaccines capable of eliciting Env-specific bNAbs^10^, a vaccine capable of eliciting high titre anti-Tat NAb might be a feasible alternative to controlling HIV replication and delaying disease onset^11^. An effective HIV vaccine should also induce robust poly-functional T cell mediated immunity (CMI) against the relatively conserved Gag proteins, viz. responses similar to those seen in long-term non-progressors^12^,^13^.

Among the several strategies that have been developed to generate mucosal HIV vaccines is the use of mucosally transmitted replication-competent viral vectors^14^. These vectors usually establish an infection that mimics a natural viral infection, efficiently deliver immunogens to mucosal antigen presenting cells (APCs), and facilitate the development of long-lasting humoral and CMI^15^ without the need for addition of adjuvants^14^. Several replication-competent viral vectors including adenovirus, poliovirus, influenza virus, poxvirus and cytomegalovirus vectors have been developed and tested as HIV vaccines, with promising results in large animal models^14^,^15^, particularly when used in heterologous prime-boost vaccination regimens.

Previously, polioviruses, which are transmitted via mucosal surfaces^16^, were studied as potential viral vectors for HIV vaccine development^16^,^17^ and vaccination with a cocktail of live recombinant polioviruses generated protective immunity against intravaginal challenge with SIVmac251 in 4/7 (57%) vaccinated macaques^17^. However, replication-competent poliovirus vectors have not advanced to human clinical trials mainly due to the high level of pre-existing vaccine-induced immunity in the community^18^ which has been shown to limit the efficacy of virus-vectored vaccines^1^. Like polioviruses, human rhinoviruses (HRVs) are classified in the *Picornaviridae* and share many characteristics, including their genome organisation and mode of transmission^19^. HRVs are transmitted via the nasal mucosa^20^ making them potential vaccine vectors to elicit mucosal immunity.

We have previously developed a series of replication-competent, genetically stable recombinant HRV serotype-A1 viruses (rHRV-Gag 1–5 and rHRV-Tat) by inserting discrete overlapping fragments of the HIV *gag* gene or the full length *tat* gene, into the junction of the genes encoding the structural proteins and the non-structural proteins (the P1/P2 junction)^21^. We have now mixed these rHRVs into a single cocktail vaccine suitable for intranasal (IN) administration, a route that has been shown to generate pan-mucosal and systemic immunity^8^. Furthermore, IN vaccination is considered to be safe, well tolerated, easily administered and inexpensive^8^, and consequently, the rHRV-Gag/Tat vaccine represents a potential cost-effective HIV vaccine candidate for use in low-income countries. Herein we describe the immune responses against Gag and Tat after vaccination of mice IN with rHRV-Gag/Tat followed by ID delivery of DNA encoding Gag and Tat, and evaluate the efficacy of this regimen after challenge with ecotropic murine leukaemia HIV (EcoHIV).

## Results

### rHRV-DNA prime-boost vaccination elicits robust CMI

**Figure 1:**
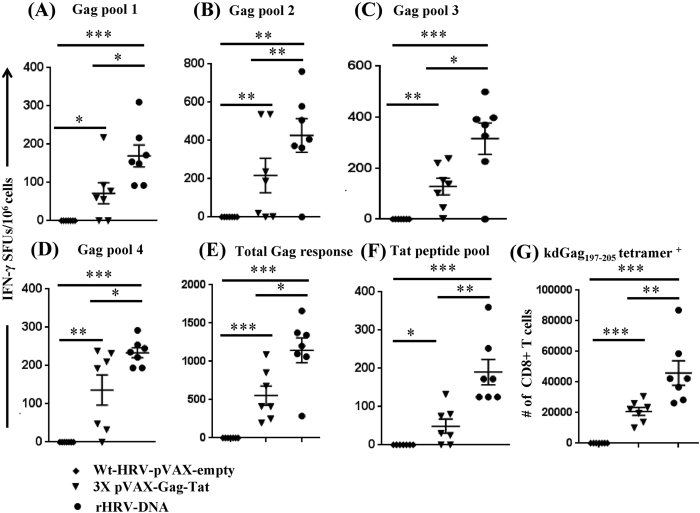
rHRV-DNA vaccination elicits robust CMI in the spleen. Mice (n = 7) received either 2 doses (containing 5 × 10^6^ TCID_50_ per dose per animal) of wild-type (wt)-HRV-A1 followed by 50 μg pVAX (vaccination control group) or 2 doses of rHRV-Gag/Tat (containing 5 × 10^6^ TCID_50_ per dose per animal) followed by a single ID dose of 50 μg of a DNA cocktail (pVAX-Gag-Tat) containing equimolar concentrations of pVAX-sTat-IMX313 and pVAX-Gag-PRF. This vaccination regimen is referred to as rHRV-DNA prime-boost vaccination. Other mice were vaccinated with 3 ID doses (50 μg/dose per animal) of pVAX-Gag-PRF/pVAX-sTat-IMX313 and referred to as 3X pVAX-Gag-Tat vaccination. Splenocytes were collected 14 days after the final dose and restimulated in duplicate with overlapping peptides representing the entire Gag protein. (**A**) Gag peptide pool 1, (**B**) Gag peptide pool 2, (**C**) Gag peptide pool 3 and (**D**) Gag peptide pool 4 in an IFN-γ ELIspot. (**E**) Total Gag responses depicted in pools 1–4. (**F**) Tat-specific responses from cells stimulated with the complete Tat peptide pool. Splenocytes were also stained with the H-2K^d^-Gag_197–205_ tetramer for 1 h at room temperature and the number of tetramer-positive CD8^+^ T cells was analyzed by flow cytometry (**G**). The data are representative of 2 independent experiments (n = 7) and are plotted as mean SFU per 10^6^ splenocytes (±SEM). Each symbol represents an individual mouse. *p < 0.05, **p < 0.01 and ***p < 0.001 (Mann–Whitney U test).

A robust CMI to HIV Gag appears to correlate with control of HIV infection in humans^12^ and non-human primates^22^,^23^. We previously showed that vaccination of mice with 3 doses of pVAX-Gag-PRF elicited broad, poly-functional Gag-specific CMI able to control EcoHIV after challenge^24^, and that vaccination with pVAX-sTat-IMX313 elicited high titre anti-Tat responses that also controlled EcoHIV infection post challenge^25^. Moreover, Tat shows adjuvant activities and the ability to broaden CTL responses without affecting Th2 responses when included together Gag- or Env-based HIV vaccines^26^,^27^. Therefore, we wished to compare the efficacy of rHRV-DNA vaccination with that of wt-HRV-A1/pVAX or 3X pVAX-Gag-Tat vaccination. Initially, we used the IFN-γ ELISpot assay (and Gag or Tat peptide pools) to compare CMI responses in splenocytes harvested 14 days after the final vaccine inoculation. This experiment showed that the Gag-specific responses to the different peptide pools ranged from 169 to 427 and 72 to 278 (mean SFU/10^6^ cells), in animals vaccinated with rHRV-DNA and 3X pVAX-Gag-Tat, respectively (Fig. 1A–D). These responses were detected in splenocytes stimulated with all 5 Gag peptide pools suggesting that each of the 5 Gag fragments encoded in the rHRV-Gag/Tat cocktail vaccine was expressed *in vivo* and thus contributed to the resultant Gag-specific responses. Splenocytes from the wt-HRV/pVAX vaccination control group showed responses which were considerably lower (<22 mean IFN-γ SFU) than those from unstimulated cells from vaccinated mice (mean IFN-γ SFU = 25), thus the net IFN-γ responses in this vaccination group is considered zero. The combined Gag response was ~2 fold higher after vaccination with rHRV-DNA compared to 3X pVAX-Gag-Tatvaccination (1147 and 554, respectively, p = 0.0175) as shown in Fig. 1E. Similarly, wt-HRV/pVAX vaccination failed to induce Tat-specific CMI responses above background (mean IFN-γ SFU = 0), but high magnitude Tat-specific CMI responses were readily detected after rHRV-DNA vaccination (mean IFN-γ SFU = 190) as shown in Fig. 1F. Furthermore, the Tat-specific CMI responses elicited by rHRV-DNA were ~3.9 fold higher than those elicited by 3X pVAX-Gag-Tat (190 compared to 49 SFU, respectively, p = 0.0041) as shown in Fig. 1F.

As IFN-γ ELISpot assays involve extensive *in vitro* stimulation of effector cells, which can cause activation-induced cell death^28^, splenocytes from immunized mice were stained with the H-2K^d^-restricted Gag_197–205_ tetramer to directly enumerate the Gag-specific CD8^+^ T cells present *in vivo*. The mean number of tetramer-positive CD8^+^ T cells following wt-HRV-pVAX vaccination was less than background from unstained cells, thus the net number of tetramer positive cells in this vaccination group is considered zero. Moreover, tetramer binding CD8^+^ T cells (mean of tetramer positive CD8^+^ T cells = 45921) could be readily detected after rHRV-DNA vaccination and these were also significantly higher than those generated following 3X pVAX-Gag-Tat vaccination (45921 vs 20814, P = 0.0023) as shown in Fig. 1G. Collectively, these results illustrate that rHRV-DNA is more effective at inducing Gag and Tat-specific CMI than wt-HRV-pVAX or 3X pVAX-Gag-Tat vaccination.

### rHRV-DNA elicits superior systemic poly-functional CMI

**Figure 2:**
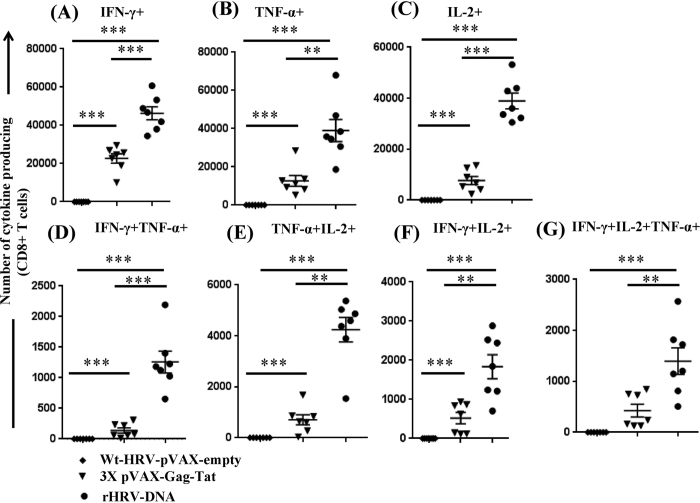
rHRV-DNA elicits superior systemic poly-functional CMI. Mice were vaccinated as described in the legend to Fig. 1 and splenocytes harvested 14 days after the final dose. The cells were stimulated for 1 h with 5 μg/ml of the H-2K^d^-restricted Gag_197–205_ immuno-dominant peptide, in the presence of protein transport inhibitor (Brefeldin A, eBiosciences) for a further 6 h and cytokine production then analyzed by flow cytometry. We gated on CD8^+^ T cells to assess the number of CD8^+^ T cells producing (**A**) IFN-γ, (**B**) TNF-α, (**C**) IL-2, (**D**) IFN-γ and TNF-α, (**E**) TNF-α and IL-2, (**F**) IFN-γ and IL-2 and (**G**) IFN-γ, TNF-α and IL-2 after stimulation.The data are representative of 2 independent experiments, plotted as mean (n = 7) ± SEM and each symbol represents an individual mouse. *p < 0.05, **p < 0.01, ***p < 0.001 and ns, p > 0.05 (Mann–Whitney U test).

T cells capable of producing multiple anti-viral cytokines (eg. IFN-γ and TNF-α) and/or survival/proliferative cytokines (eg. IL-2) i.e. poly-functional T cells, are desirable outcomes for HIV-1 vaccine development as the presence of these cells appears to correlate with improved control of HIV infection in humans^12^,^29^ and SIV infection in non-human primates^22^,^23^. The data showed that K^d^Gag_197–205_-specific CD8^+^ T cells from all rHRV-DNA vaccinated animals responded to vaccination by producing IL-2, IFN-γ or TNF-α, whereas responses from wt-HRV-A1/pVAX vaccinated animals did not surpass background from unstimulated cells (Fig. 2A–C). Furthermore, the number of CD8^+^ T cells producing IFN-γ, IL-2 or TNF-α was ~2 fold (mean 46318 vs 22679, *P* = 0.0006), ~5 fold (38928 vs 7712, *P* = 0.0006) and ~3 fold (39007 vs 12738, *P* = 0.0012) higher following rHRV-DNA than after 3X pVAX-Gag-Tat vaccination, respectively (Fig. 2A–C).

The number of Gag-specificCD8^+^ T cells that concurrently produced at least 2 cytokines (poly-functional CD8^+^ T cells) following K^d^Gag_197–205_ peptide stimulation was also determined. Vaccination with rHRV-DNA resulted in ~9-fold more cells producing IFN-γ and TNF-α than 3X pVAX-Gag-Tat vaccination (1259 vs 140, *P* = 0.0006) as shown in Fig. 2D. Similarly, the number of cells that produced IL-2 and TNF-α was ~6 fold higher in rHRV-DNA vaccinated mice (4240 vs 707, *P* = 0.0012) as shown in Fig. 2E, while the number of cells producing IFN-γ and IL-2 was ~2.5 fold (1836 vs 523, *P* = 0.0041) higher (Fig. 2F). The number of triple cytokine- (IFN-γ, IL-2 and TNF-α) producing CD8^+^ T cells was ~3.3 fold higher after rHRV-DNA vaccination (1398 vs 427, *P* = 0.0070) as shown in Fig. 2G.

We then examined the number of CD8^+^ cells with an effector memory T cell (T_EM_) phenotype. Effector memory is vital to establish antigen-specific recall responses^22^,^30^ and is required for an effective HIV vaccine. As T_EM_ are marked by CD44 expression^31^, we determined the number of CD44^hi^CD8^+^ T cells which synthesised cytokines after splenocytes were stimulated with the K^d^Gag_197–205_ peptide.

The number of CD44^hi^CD8^+^ T cells that produced IFN-γ, TNF-α or IL-2 was 3–4 fold higher in the rHRV-DNA-than in the 3X pVAX-Gag-Tat vaccination group (Supplementary Fig. S1A–C). Although the number of double and triple positive CD44^hi^CD8^+^ T cells was modest after rHRV-DNA or 3X pVAX-Gag-Tat vaccination, it was significantly higher in the rHRV-DNA group (Supplementary Fig. S1D–G, respectively). In summary, the data also illustrate that rHRV-DNA is more effective at inducing poly-functional Gag-specific CD8^+^ T cell responses in the spleen than wt-HRV-pVAX or 3X pVAX-Gag-Tat vaccination.

### rHRV-DNA vaccination elicits superior poly-functional CMI in the gut mucosa

**Figure 3:**
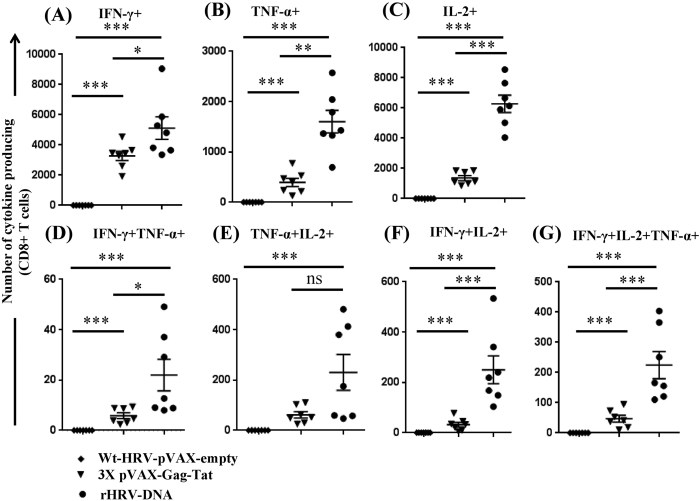
rHRV-DNA vaccination elicits superior poly-functional CMI in the gut mucosa. Mice were vaccinated as described in the legend to Fig. 1 and lymphocytes prepared from mesenteric lymph nodes harvested 14 days after the final dose. The lymphocytes were stimulated for 1 h with 5 μg/ml of the H-2K^d^-restricted Gag_197–205_ immuno-dominant peptide and then cultured in the presence of Brefeldin A for a further 6 h. Cytokine production was analyzed by flow cytometry. We gated on CD8^+^ T cells to assess the number of CD8^+^ T cells producing (**A**) IFN-γ, (**B**) TNF-α, (**C**) IL-2, (**D**) IFN-γ and TNF-α, (**E**) TNF-α and IL-2, (**F**) IFN-γ and IL-2 and (**G**) IFN-γ, TNF-α and IL-2 after stimulation. The data are representative of 2 independent experiments, plotted as mean (n = 7) ± SEM and each symbol represents an individual mouse. *p ≤ 0.05, **p ≤ 0.01 and ***p ≤ 0.001 (Mann–Whitney U test).

The vast majority of CD4^+^ T cell death during acute HIV-1 infection occurs in the gut and thus poly-functional CMI responses at the gut mucosa are likely to significantly reduce CD4^+^ T cell death^32^. Vaccination with wt-HRV-A1/pVAX failed to induce K^d^Gag_197–205_-specific CD8^+^ T cell responses above background from unstimulated lymphocytes prepared from mesenteric lymph nodes, whereas high numbers of Gag-specific cells were readily detected following rHRV-DNA vaccination (Fig. 3A–G). Furthermore, the number of mono-functional (Fig. 3A–C) and poly-functional cells (Fig. 3D–G) generated after rHRV-DNA was significantly higher than that after 3X pVAX-Gag-Tat vaccination. The number of poly-functional T_EM_ cells derived from the mesenteric lymph nodes produced following rHRV-DNA or 3X pVAX-Gag-Tat vaccination was generally modest, although rHRV-DNA appeared to be more effective than 3X pVAX-Gag-Tat (Supplementary Fig. S2). Overall, consistent with the splenocyte data, rHRV-DNA induced poly-functional Gag-specific CD8^+^ T cell responses in the gut more effectively than wt-HRV-pVAX or 3X pVAX-Gag-Tat vaccination.

### rHRV-DNA vaccination elicits superior Tat-specific humoral responses

**Figure 4:**
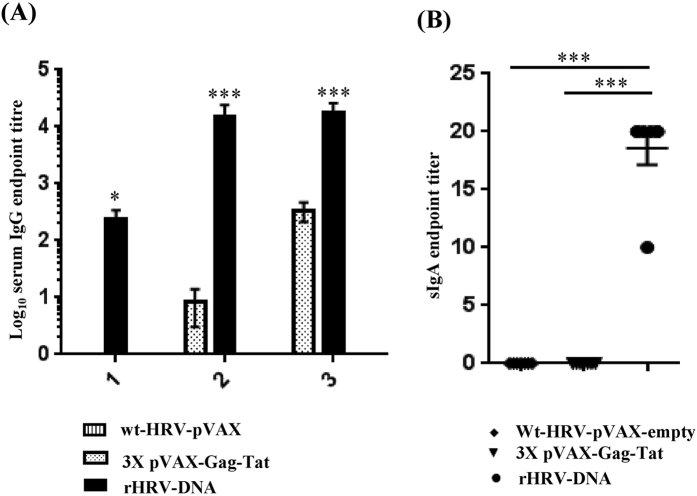
rHRV-DNA vaccination elicits superior Tat-specific humoral responses. Mice were vaccinated as described in the legend to Fig. 1. Blood and CVL samples were collected 1 day before each vaccination and 14 days after the final dose. (**A**) ELISA results showing serum log_10_ anti-Tat IgG titres. (**B**) ELISA results showing anti-Tat sIgA titres. Data are plotted as mean (n = 7) ± SEM and are representative of 2 independent experiments. *p ≤ 0.05 and ***p ≤ 0.001 (Mann–Whitney U test).

High titre Tat-specific serum IgG appears to correlate with control of HIV infection in humans^11^,^33^ and SIV in macaques^34^,^35^. Therefore, we compared the titres of Tat-specific IgG generated after vaccination with wt-HRV-A1/pVAX, rHRV-DNA or 3X pVAX-Gag-Tat. Tat-specific IgG was detected in serum samples from all vaccinated mice (except in the wt-HRV-A1/pVAX group) with mean reciprocal endpoint titres ranging from 270 to 65610 in the rHRV-DNA vaccinated group and from 30 to 810 in the 3X pVAX-Gag-Tat vaccinated group (Fig. 4A). In the rHRV-DNA vaccinated group, anti-Tat IgG was detectable in samples after the first rHRV dose with a mean titre of 236, but was undetectable in the 3X pVAX-Gag-Tat vaccinated group at this time point. After the 2^nd^ dose, the mean anti-Tat antibody titre in serum from the rHRV-DNA vaccinated group increased by ~65 fold (from 236 to 15312) and was considerably higher than that in serum from the 3X pVAX-Gag-Tat group (mean titer = 9). Administration of a single DNA booster dose increased the IgG titres by ~1.2 fold (from 15312 to 17704) in the rHRV-DNA group and by ~38 fold (from 9 to 338) in the 3X pVAX-Gag-Tat vaccinated group. The differences in serum IgG titres were statistically significant at each time point between the two vaccinated groups (Fig. 4A). The OD generated by serum from wt-HRV/pVAX vaccinated animals was lower than the cut-off, thus the antibody titre in this vaccination group was considered to be zero.

We also examined CVL samples from vaccinated animals for Tat-specific mucosal sIgA, as the presence of antigen-specific sIgA in blood or at the mucosa appears to correlate with protection in humans^36^,^37^. We detected modest levels of sIgA (mean titre 20) in CVL samples collected after the DNA boost from all mice in the rHRV-DNA group, but not from mice in the wt-HRV/pVAX or 3X pVAX-Gag-Tat vaccinated group (Fig. 4B). Most surprisingly, sIgA was not detected in samples prior to the DNA boost, indicating that this was required for the rHRV-Gag/Tat vaccine to induce detectable mucosal sIgA. On the whole, the results described above indicate that rHRV-DNA is more effective at inducing robust Gag and Tat-specific CMI as well as Tat-specific humoral immune responses at the mucosa than wt-HRV-pVAX or 3X pVAX-Gag-Tat vaccination.

### rHRV-DNA controls the EcoHIV viral load post-challenge

EcoHIV is a murine HIV challenge model and has been used previously to evaluate the efficacy of candidate HIV vaccines^24^,^38^. However, there is no documented evidence that EcoHIV can infect mice via the intravaginal route which accounts for a majority of HIV transmissions^6^, and thus the IP route is a convenient route to successfully deliver the virus^24^,^38^. EcoHIV encodes the complete range of HIV proteins, including Tat, from the HIV-1 Clade B NL4-3 strain, except for the HIV gp120 which was replaced with gp80 from the murine leukaemia virus^39^. The virus spreads from the primary site of infection to the spleen and the brain in a manner reminiscent of HIV infection^39^.

**Figure 5:**
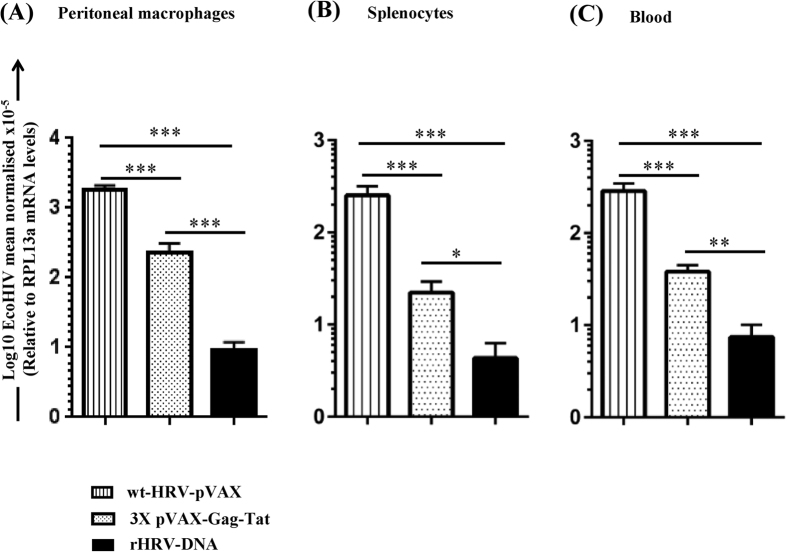
rHRV-DNA vaccination controls EcoHIV viral load post-challenge. Mice were vaccinated with rHRV-DNA, wt-HRV- pVAX or 3X pVAX-Gag-Tat at 2 week intervals and then challenged with 1.5 μg p24 of EcoHIV/NL4-3 10 days post final vaccination. The viral load in PECs, splenocytes and blood was determined by qRT-PCR; EcoHIV RNA levels in (**A**) PECs, (**B**) splenocytes and (**C**) blood 7 days post challenge. EcoHIV mRNA levels were normalised to RPL13a mRNA and the data represent the mean (n = 7) ± SEM. *p ≤ 0.05, **p ≤ 0.01 and ***p ≤ 0.001 (Mann–Whitney U test).

Consequently, we examined the protective efficacy of wt-HRV/pVAX, rHRV-DNA or 3X pVAX-Gag-Tat vaccination against EcoHIV challenge. All animals were challenged 10 days after the final vaccination, then culled 7 days later and splenocytes, PECs and peripheral blood samples collected to quantify the viral load (VL) by qRT PCR. The results were normalised to the RPL13a house-keeping gene as described previously^24^,^38^. Vaccination with rHRV-DNA or 3X pVAX-Gag-Tat significantly reduced the EcoHIV VL by ~204 fold (*p* = 0.0006) and ~8 fold (*p* = 0.0006) in PECs (Fig. 5A), ~62 fold (*p* = 0.0006) and ~11 fold (*p* = 0.0006) in splenocytes (Fig. 5B), and by ~40 fold (*p* = 0.0006) and ~7.5 (*p* = 0.0006) in blood (Fig. 5C), respectively, compared to vaccination with wt-HRV/pVAX. Furthermore, rHRV-DNA vaccination showed superior control of EcoHIV infection compared to 3X pVAX-Gag-Tat vaccination and reduced the VL by ~25 fold (*p* = 0.0006) in PECs, ~5.5 fold (*p* = 0.0286) in splenocytes and by ~5.4 fold (*p* = 0.0035) in peripheral blood as shown in Fig. 5A–C. Taken together, the data suggest that the rHRV-DNA regimen can also be used to achieve effective virologic control of EcoHIV-1 infection.

## Discussion

In this study, we report that a vaccination regimen comprised of a cocktail of replication-competent rHRVs encoding HIV Gag and Tat (rHRV-Gag/Tat) followed by a DNA vaccine encoding the same proteins (pVAX-Gag-Tat), elicited robust Gag-specific CMI in the spleen and gut mucosa, and high titer anti-Tat serum IgG and sIgA in the vagina of vaccinated mice. Furthermore, the vaccine-elicited immune responses controlled infection in a mouse EcoHIV challenge model. Intranasal (IN) vaccination generates pan-mucosal and systemic immunity as described previously^8^ and our data provide new support to consider IN vaccination as a method to induce anti-HIV immunity at the mucosa and systemically. It is not possible to directly compare the immunogenicity and efficacy of rHRV-Gag/Tat vaccination to that reported previously by Crotty *et al.* using the recombinant Sabin poliovirus-based HIV vaccines in macaques due to differences in animal models and challenge viruses used^17^. However, our data confirm that, similar to Sabin poliovirus-based vaccines, IN administration of the rHRV-Gag/Tat vaccine in a heterologous prime-boost strategy (rHRV-DNA) elicited systemic and mucosal HIV-specific immune responses that significantly controlled EcoHIV infection.

Heterologous prime-boost vaccination has been shown to generate superior CMI and humoral immunity compared with homologous prime-boost vaccination^40^. Similarly, our data showed that the rHRV-DNA regimen elicited poly-functional Gag-specific CD8^+^ T cells more effectively than wtHRV-A1/pVax or 3X pVAX-Gag-Tat vaccination at the mucosa (Fig. 3 and Supplementary Fig. S2) and systemically (Figs 1 and 2 and Supplementary Fig. S1). The appearance of high quality Gag- and Tat-specific CMI responses appears to correlate with long-term HIV control in humans^12^,^13^ and SIV in non-human primates^22^,^41^,^42^, thus our data suggest that the rHRV-DNA regimen might control SIV when tested in macaques or HIV-1 infections in humans. In addition to robust CMI, rHRV-DNA induced higher Tat-specific serum IgG and mucosal sIgA titres than wtHRV-A1/pVax or 3X pVAX-Gag-Tat vaccination (Fig. 4A), additional markers which appear to correlate with control of HIV in humans^11^,^33^ and SIV in animals^34^,^43^. Although we did not assess functionality of the Tat-specific antibodies in terms of anti-Tat neutralization, a previous study conducted in our laboratory indicated that intradermal vaccination with pVAX-sTat-IMX313, one of the vaccines used in the regimens of this study, elicited antibodies with Tat neutralization activity that correlated with control of EcoHIV^25^. Surprisingly, Tat-specific sIgA was detected in CVL samples from rHRV-DNA vaccinated animals only after the DNA boost (Fig. 4B). The reason for this unexpected result remains unclear. However, sIgA was not detected in any animals vaccinated 3X pVAX-Gag-Tat at any time point; this was not surprising as we have previously shown that a minimum of 5 doses is required for a Tat DNA vaccine to elicit detectable mucosal sIgA^25^.

Although other studies generally administer recombinant viruses as a boost following a DNA prime^40^,^44^,^45^, our results are consistent with studies which have shown that administering a recombinant virus followed by a DNA^40^ or protein vaccine^46^ in a heterologous prime-boost vaccination strategy elicits high titre immune responses. Moreover, a boost with a DNA vaccine following rHRV-Gag/Tat prime is likely to elicit robust HIV-specific immune responses at the mucosa and systemically in vaccinated humans. Regular boosting with DNA may also be feasible and offers the advantage that anti-vector responses will not accumulate over time and prevent further boosting, as would be the case for a recombinant virus-based vaccine.

Tat shows potent adjuvanticity through its ability to dimerize and promote maturation and activation of monocyte-derived dendritic cells^47^, to generate and present sub-dominant MHC-1 epitopes by modifying the proteolytic activity of the immunoproteosome^48^, and induce high levels of surface MHC-1 peptide expression. Although we did not directly investigate the effect of including Tat in the rHRV-DNA vaccination regimen, based on this body of evidence, we speculate that Tat enhanced and broadened the overall Gag-specific T cell responses in addition to inducing Tat specific responses. To further support our hypothesis, Gavioli *et al.* demonstrated previously that including Tat in a multi-antigenic vaccine enhanced the magnitude and broadened Gag- and Env-specific T cell responses without affecting the quality of the Th2 responses^27^. Furthermore, the inclusion of Tat in a candidate vaccine against Herpes Simplex Virus type 1 (HSV-1) enhanced the induction of broad, high magnitude effector memory HSV-1 specific CD 8 T cell responses^49^ that completely protected mice after a lethal intravaginal challenge with wild type HSV-1^49^,^50^ HSV-1. Collectively, this evidence supports the inclusion of Tat as a component of a multi antigenic vaccine and implies that the rHRV-DNA vaccination regimen reported here, that contains Tat and Gag as immunogens, will most likely be highly immunogenic.

As robust immune responses do not necessarily indicate protection against virus challenge^51^,^52^, we evaluated the efficacy of rHRV-DNA vaccination using a murine HIV challenge model, EcoHIV^39^. The rHRV-DNA regimen resulted in effective control of the EcoHIV infection (Fig. 5A,B). However, it is unclear if humoral immunity and/or CMI were responsible for this control and additional experiments will be necessary to address this issue. Nevertheless, the robust Gag-specific CMI and high titer anti-Tat responses are thought to offer protective benefits against HIV in long-term non-progressors^11^,^12^ and non-human primates^23^,^34^, and thus it is possible that both Gag-CMI and anti-Tat responses contributed.

In conclusion, we demonstrate for the first time that a live recombinant HRV vaccine encoding HIV proteins when administered in a heterologous vaccination regimen involving priming with virus followed by a single DNA boost is highly immunogenic in mice and that the vaccine-induced immune responses control EcoHIV infection post challenge. The data presented in this study have important ramifications for the development of effective mucosal HIV-1 vaccines in the future.

## Methods

### Recombinant HRV-Gag/Tat production and purification

We have previously described the production of rHRV-Gag/Tat^21^. Briefly, five overlapping fragments representing the full length HIV *gag* or the *tat* gene were individually inserted into the P1/P2 junction in a replication-competent HRV A1 cDNA^53^ to generate 5 rHRVs each encoding a discrete, overlapping fragment of the Gag protein and one rHRV encoding the full length Tat protein. Each recombinant virus was produced by transfecting H1-Hela cells with *in vitro* transcribed viral RNA generated with the TransIT^®^-mRNA transfection kit (Mirus) following the manufacturer’s instructions. The individual rHRVs were purified and titrated as described previously^21^ then combined into a cocktail (rHRV-Gag/Tat) and re-titrated to ensure that the vaccination dose contained a uniform virus concentration.

### DNA vaccines

Plasmids encoding HIV Gag and perforin (PRF) (pVAX-Gag-PRF) or HIV Tat fused to the oligomerisation domain of the C4b-p (pVAX-sTat-IMX313) have been described recently^24^,^25^. Briefly, the HIV *gag* and PRF genes were inserted downstream of the CMV and SV40 promoters, respectively, to generate pVAX-Gag-PRF^24^. To generate pVAX-sTat-IMX313, the human tissue plasminogen activator leader sequence and the oligomerisation domain of C4b-p (IMX313) were introduced at the N- and C-termini respectively of the HIV *tat* gene^25^.

### Animals and immunisations

Female 6–8 week old BALB/c mice were obtained from the University of Adelaide Laboratory Animal Services and maintained under PC2 conditions in individually ventilated cages fitted with a HEPA filter. All experimental animal protocols were approved by the Women’s and Children’s Health Network (project licence number AE783-02-2012), the South Australia Pathology Animal Ethics committee (project licence number 29-15) and the University of Adelaide Animal Ethics Committee (project licence number M-2012-201) under the regulations and guidelines of “Australian Code of Practice for the Care and Use of Animals for Scientific Purposes” issued by the Australian National Health and Medical Research Council (NHMRC). Vaccinations were performed at two week intervals via the IN route after 2% isofluorane anaesthesia for the rHRV-Gag/Tat cocktail andwild-type (wt)-HRV-A1 as described^54^ or via the intradermal (ID) route for the DNA vaccines as described^24^. We have used intradermal vaccination for DNA vaccines for some time because this route has a higher frequency of dendritic cells (DC) and other antigen presenting cells than the intramuscular route^55^,^56^. As the efficacy of vaccination is dependent on uptake, processing and presentation of the immunogen by DC, then we and others believe that this route is more effective.

Mice (n = 7) received either 2 doses (containing 5 × 10^6^ TCID_50_ per dose per animal) of wt-HRV-A1 followed by 50 μg pVAX (vaccination control group, referred to hereafter as wt-HRV-A1/pVax) or 2 doses of the rHRV-Gag/Tat cocktail (containing 5 × 10^6^ TCID_50_ per dose per animal) followed by a single ID dose of 50 μg of a DNA cocktail (pVAX-Gag-Tat), containing equimolar concentrations of pVAX-sTat-IMX313 and pVAX-Gag-PRF. The latter will be referred to hereafter as rHRV-DNA prime-boost vaccination. Another group of mice received 3 ID doses (50 μg of a DNA cocktail per dose) of pVAX-Gag-Tat and this homologous prime-boost vaccination strategy will be referred to hereafter as 3X pVAX-Gag-Tat vaccination. Blood and cervical vaginal lavage (CVL) samples were collected one day prior to each vaccination and 14 days after the final vaccination, when the mice were euthanized, and examined for anti-Tat antibody responses. Splenocytes and lymphocytes from the mesenteric lymph nodes (mesenteric lymphocytes) were also harvested at this time point for analysis of CMI.

### Enzyme-linked immunosorbent spot assay (ELISpot)

ELISpots were performed to determine the breadth and magnitude of HIV-specific CMI in splenocytes from vaccinated mice. A panel of 15–19 mer overlapping peptide pools spanning the entire Gag and Tat proteins was obtained from the NIH AIDS reagent bank (Germantown, MD, USA). Mouse interferon (IFN)-γ ELISpot was performed on RBC-depleted splenocytes that were re-stimulated for 36 h with 4 μg/ml of 5 Gag peptide pools or a Tat peptide pool as we described previously^24^,^38^. Briefly, multiscreen-IP HTS plates (Millipore) were coated with anti-mouse IFNγ (clone AN18, MabTech) and secreted IFNγ detected with anti-mouse IFNγ-biotin (clone R4-6A2, MabTech). Developed spots were counted automatically using an ELISpot reader (AID GmbH, Germany). The number of spots (spot forming units-SFUs) in unstimulated splenocytes was subtracted from the number in peptide-stimulated cells to generate the net Gag or Tat response.

### Intracellular cytokine staining (ICS) and flow cytometry

Multi-colour ICS was performed on RBC-depleted splenocytes and mesenteric lymphocytes to determine IFN-γ, interleukin (IL)-2, and tumor necrosis factor (TNF)-α production from Gag-specific CD8^+^ T cells as described previously^24^,^38^. Briefly, the cells were stimulated for 1 h with 5 μg/ml of the H-2K^d^-restricted Gag_197–205_ (AMQMLKETI) immunodominant peptide^57^ (China peptides, China) then cultured in the presence of protein transport inhibitor (Brefeldin A, eBiosciences) for a further 6 h. Staining was performed using fluorescence-activated cell sorting (FACS) Cytofix/Cytoperm buffer (BD Biosciences) with antibodies specific to mouse antigens CD8α (allophycocyanin (APC)-efluor780 conjugated), CD44α (APC conjugated), IL-2 (peridinin chlorophyll 5.5 conjugated), IFN-γ (fluorescein isothiocyanate (FITC) conjugated) and TNF-α (phycoerythrinconjugated) (BD Biosciences). The cells were analysed on a BD FACS Cantoand the results analysed with FlowJo X.0.7 software (Ashland, OR) using the gating strategy shown in Supplementary Figs S3–S6. The number of cells producing cytokines without prior stimulation was subtracted from the number in peptide-stimulated cells to generate the net Gag response.

### H-2K^d^- Gag_197–205_ tetramer and antibody staining

RBC-depleted splenocytes were initially stained with the APC-conjugated H-2K^d^-restricted Gag_197–205_ (AMQMLKETI) immunodominant peptide tetramer (Biomolecular Resource Facility, John Curtin School of Medical Research, Australian National University, Canberra, Australia) for 1 h at room temperature, washed twice in phosphate-buffered saline (PBS) and then re-stained with FITC-conjugated anti-mouse CD8α antibody (BD Biosciences). The cells were analysed on a BD FACS Canto and the results analysed with FlowJo X.0.7 software using the gating strategy shown in Supplementary Fig. S7. The number of unstained tetramer positive cells was subtracted from the number in tetramer-stained cells to generate the net tetramer positive cells.

### Enzyme-linked immunosorbent assay (ELISA)

Serum and CVL samples from mice were analyzed for anti-Tat antibodies by indirect ELISA as we described previously^25^. Briefly, Maxisorp plates (Corning Sigma-Aldrich) were coated with 500 ng of purified Tat protein in carbonate-bicarbonate buffer, blocked with 2% w/v bovine serum albumin (BSA) in PBS and then serial dilutions of serum and CVL samples added. Bound antibodies were detected using horseradish peroxidase (HRP)-conjugated goat anti-mouse immunoglobulin (Ig)G (GE Healthcare Life Sciences, USA) or anti-mouse IgA (Life Technologies) and the optical density (OD) read at 492 nm. Endpoint titres were determined as the reciprocal of the highest serum or CVL sample dilution with an OD reading above the cut-off, set as twice the standard deviation (SD) above the mean OD of serum samples from naïve mice.

### EcoHIV/NL4-3 challenge

EcoHIV preparation, titration and challenge have been described previously^24^,^38^,^39^. Vaccinated mice were challenged via the intra-peritoneal (IP) route with EcoHIV equivalent to 1.5 μg p24, then spleen, blood and peritoneal exudate cells (PECs) collected 7 days post challenge and examined by qRT-PCR as described previously^24^,^58^. Results were normalised to RPL13a mRNA levels after examining primer efficiency using the ΔΔCT (threshold cycle) quantification method^59^.

### Statistical analysis

Data presented as mean ± standard error of the mean (SEM) were generated with GraphPad Prism version 6 (GraphPad Software, La Jolla, CA, USA). Non-parametric Kruskal-Wallis test was used to compare the difference between multiple vaccine groups. If this showed significant differences, then the Mann-Whitney U test was performed to compare differences between each vaccine group, independently. Statistical significance was determined using the Mann-Whitney U test; *p* < 0.05 was considered significant and *p* > 0.05 was considered non-significant.

## Additional Information

**How to cite this article**: Tomusange, K. *et al.* Mucosal vaccination with a live recombinant rhinovirus followed by intradermal DNA administration elicits potent and protective HIV-specific immune responses. *Sci. Rep.* **6**, 36658; doi: 10.1038/srep36658 (2016).

**Publisher’s note**: Springer Nature remains neutral with regard to jurisdictional claims in published maps and institutional affiliations.

## Supplementary Material

Supplementary Information
