# Supplementary material for: Mucosal vaccination with a live recombinant rhinovirus followed by intradermal DNA administration elicits potent and protective HIV-specific immune responses
Source: Sci Rep. 2016 Nov 17;6:36658. doi: 10.1038/srep36658 (PMC5113119; doi:10.1038/srep36658)
Supplement: Supplementary Information [file srep36658-s1.pdf]

Mucosal vaccination with a live recombinant rhinovirus followed by intradermal DNA administration elicits potent and protective HIV-specific immune responses

Khamis Tomusange[1], Danushka Wijesundara[1], Jason Gummow[1], Steve Wesselingh[2], Andreas Suhrbier[3], Eric J. Gowans[1] and Branka Grubor-Bauk[1#]

[1]Virology Laboratory, Basil Hetzel Institute, Discipline of Surgery, University of Adelaide, Adelaide, South Australia, Australia.

[2]South Australian Health and Medical Research Institute, Adelaide, South Australia, Australia

[3]QIMR Berghofer Medical Research Institute, Brisbane, Queensland, Australia

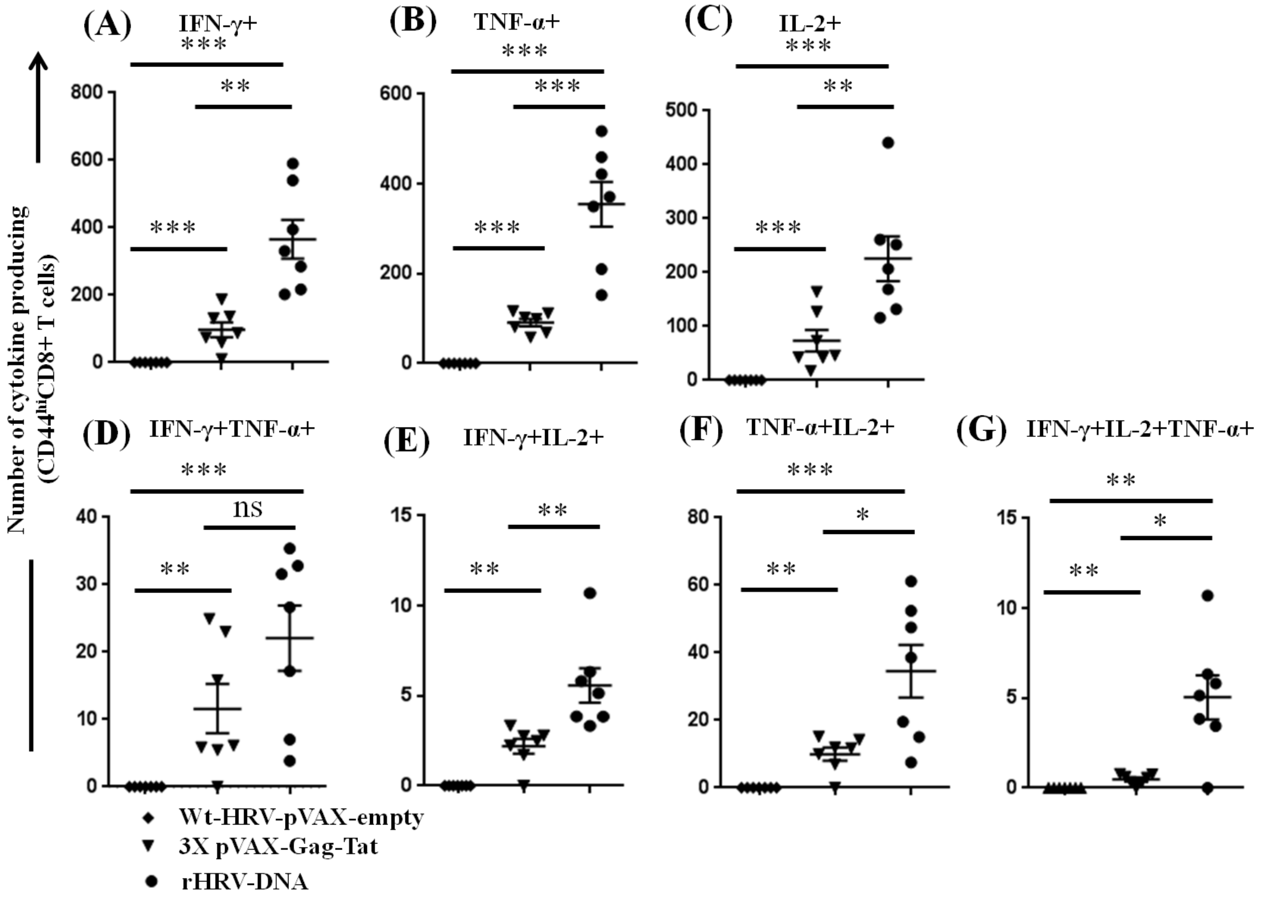

Supplementary Fig S1. rHRV-DNA vaccination elicits superior Gag-specific effector memory CMI in the spleen. Mice were vaccinated as described in the legend to figure 1 and splenocytes harvested 14 days after the last dose. Splenocytes were stimulated for 1 h with 5 µg/ml of the H-2K^{d}-restricted Gag$_{197-205}$ immuno-dominant in the presence of protein transport inhibitor (Brefeldin A, eBiosciences) for a further 6h.Cytokine production was analyzed by flow cytometry. We gated on memory CD8^{+} T cells to assess the number of CD44^{hi}CD8^{+} T cells producing (A) IFN-γ, (B) TNF-α, (C) IL-2, (D) IFN-γ and TNF-α, (E) IFN-γ and IL-2, (F) TNF-α and IL-2 and (G) IFN-γ, TNF-α and IL-2 after stimulation. The data are representative of 2 independent experiments, plotted as mean (n = 7)± SEM and each symbol represents an individual mouse. * $p \leq 0.05$, ** $p \leq 0.01$, *** $p \leq 0.001$ and ^{ns} $p > 0.05$ (Mann–Whitney U test).

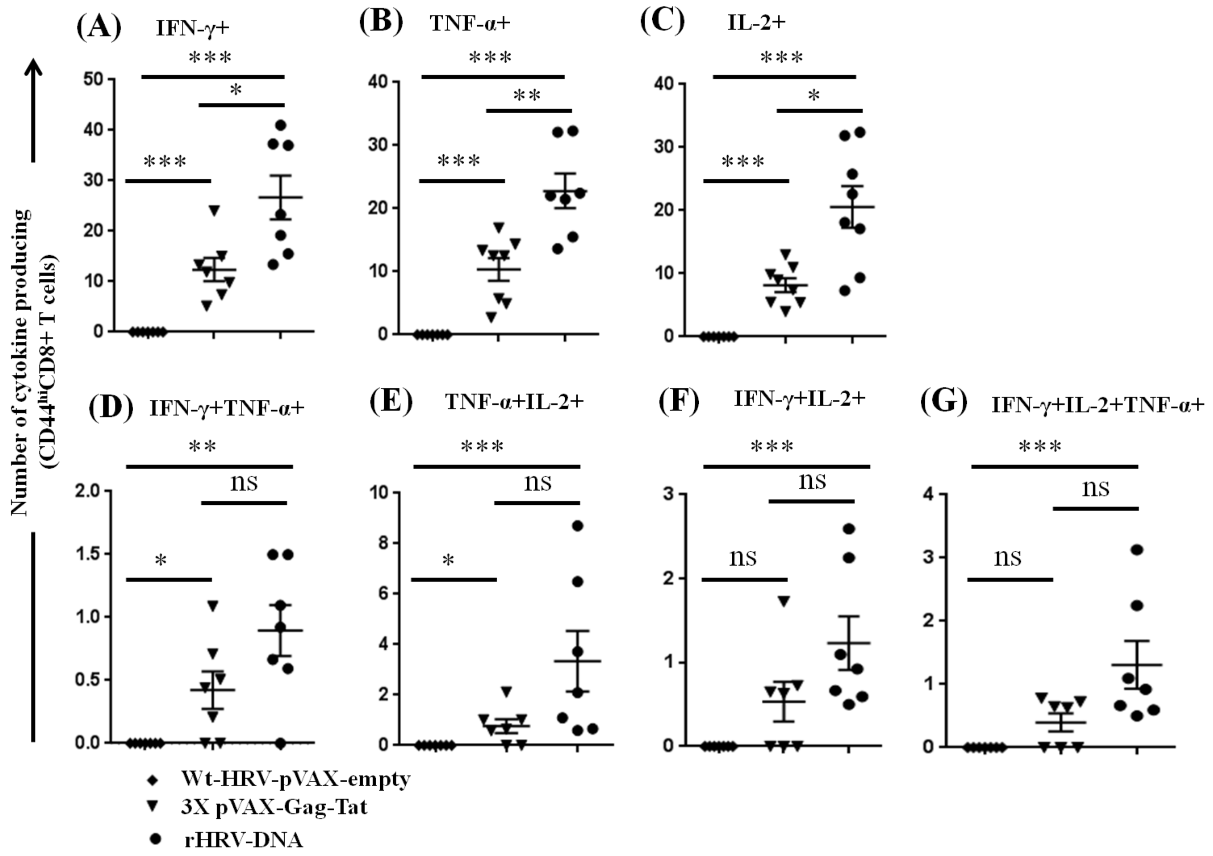

Supplementary Fig S2. rHRV-DNA vaccination elicits a robust Gag-specific effector memory CMI at the gut mucosa. Mice were vaccinated as described in the legend to figure 1and lymphocytes from mesenteric lymph nodes harvested 14 days after the final dose. The lymphocytes were stimulated for 1 h with 5 µg/ml of the H-2K^{d}-restricted Gag$_{197\text{-}205}$ immuno-dominant peptide in the presence of Brefeldin A for a further 6 h.Cytokine production was analyzed by flow cytometry. We gated on memory CD8^{+} T cells to assess the number of CD44^{hi}CD8^{+} T cells producing (A) IFN-γ, (B) TNF-α, (C) IL-2, (D) IFN-γ and TNF-α, (E)TNF-α and IL-2, (F) IFN-γ and IL-2and (G) IFN-γ, TNF-α and IL-2 after stimulation. The data are representative of 2 independent experiments and are plotted as mean (n = 7) ± SEM and each symbolrepresents an individual mouse. * $p \leq 0.05$ and ** $p \leq 0.01$ (Mann–Whitney U test).

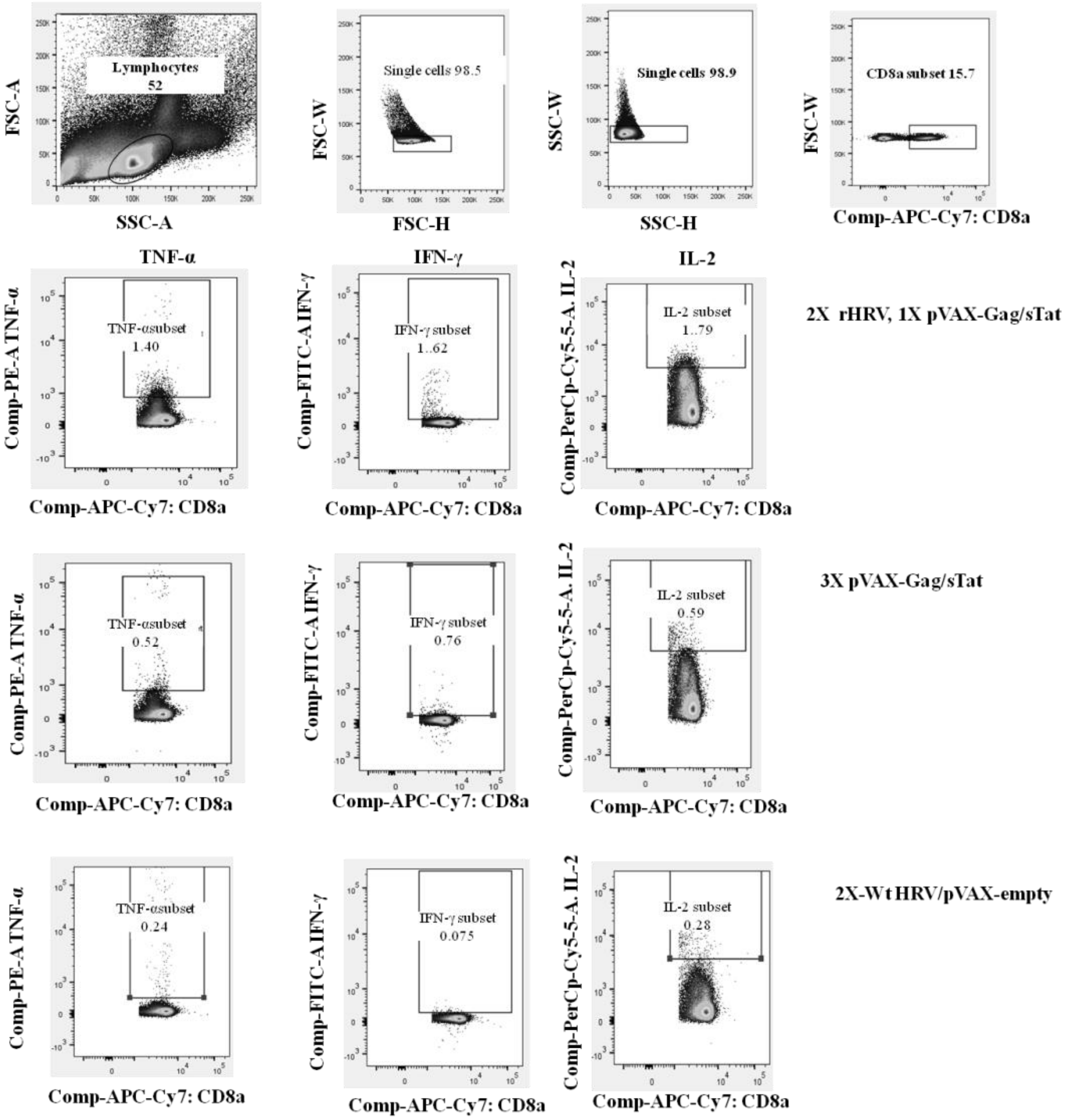

Supplementary Fig S3. Gating strategy used to detect polyfunctional CD8^{+}T cells in the spleen. Mice were vaccinated as described in the legend to figure 1and lymphocytes from mesenteric lymph nodes harvested 14 days after the final dose. Cytokine profiles were determined using flow cytometry. Splenocytes were gated on the lymphocyte population, followed by doublet discrimination, and then gated on CD8^{+} T cells to assess the frequency of IFN-γ, TNF-α and IL-2. Representative plots for IFN-γ, TNF-α and IL-2 positive cells are shown.

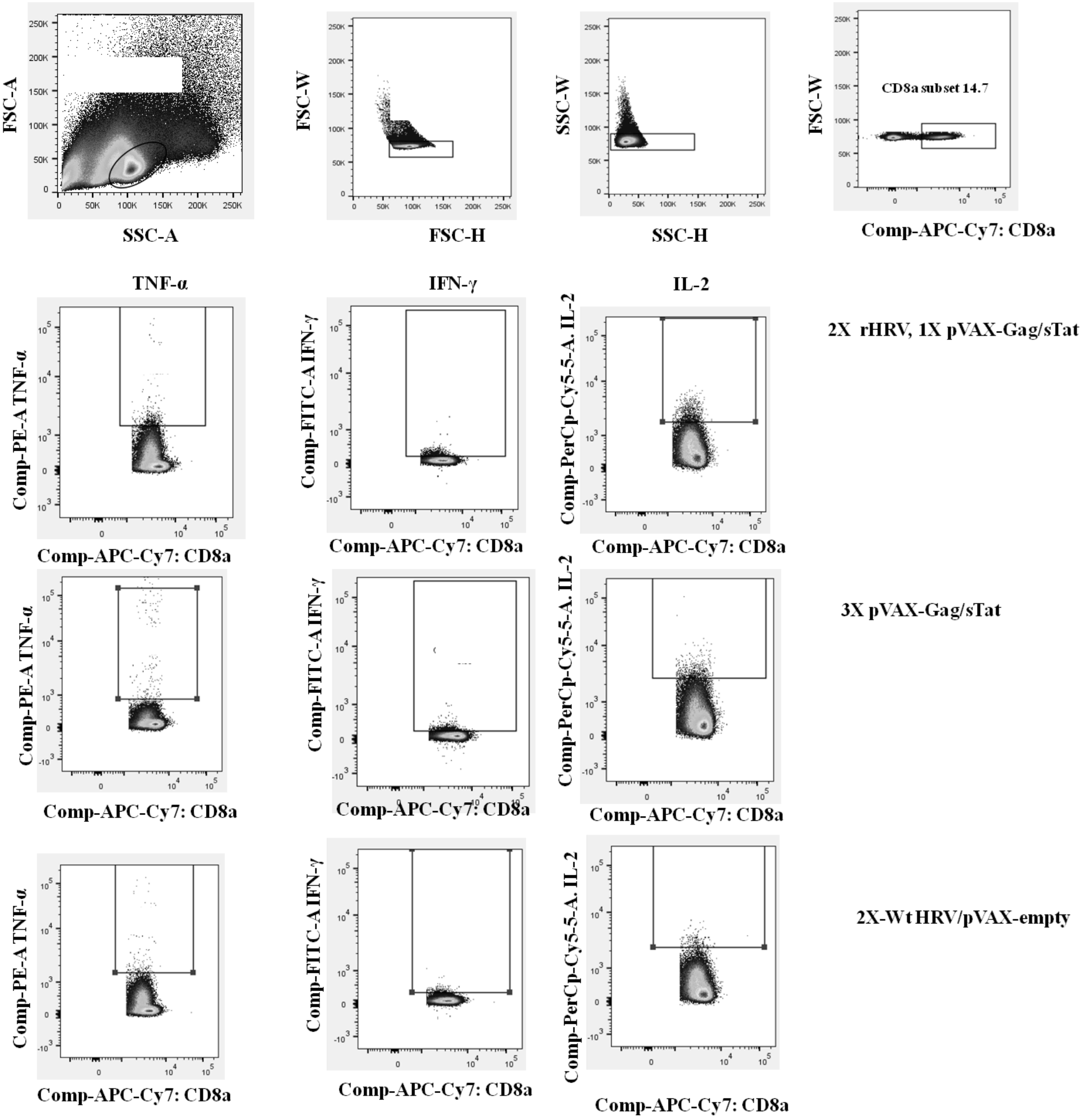

Supplementary Fig S4. Gating strategy used to detect polyfunctional CD8$^+$T cells in the gut. Mice were vaccinated as described in the legend to figure 1and lymphocytes from mesenteric lymph nodes harvested 14 days after the final dose. Mesenteric lymphocytes were gated on the lymphocyte population, followed by doublet discrimination, and then gated on CD8$^+$ T cells to assess the frequency of IFN-γ, TNF-α and IL-2. Representative plots for IFN-γ, TNF-α and IL-2 positive cells are shown.

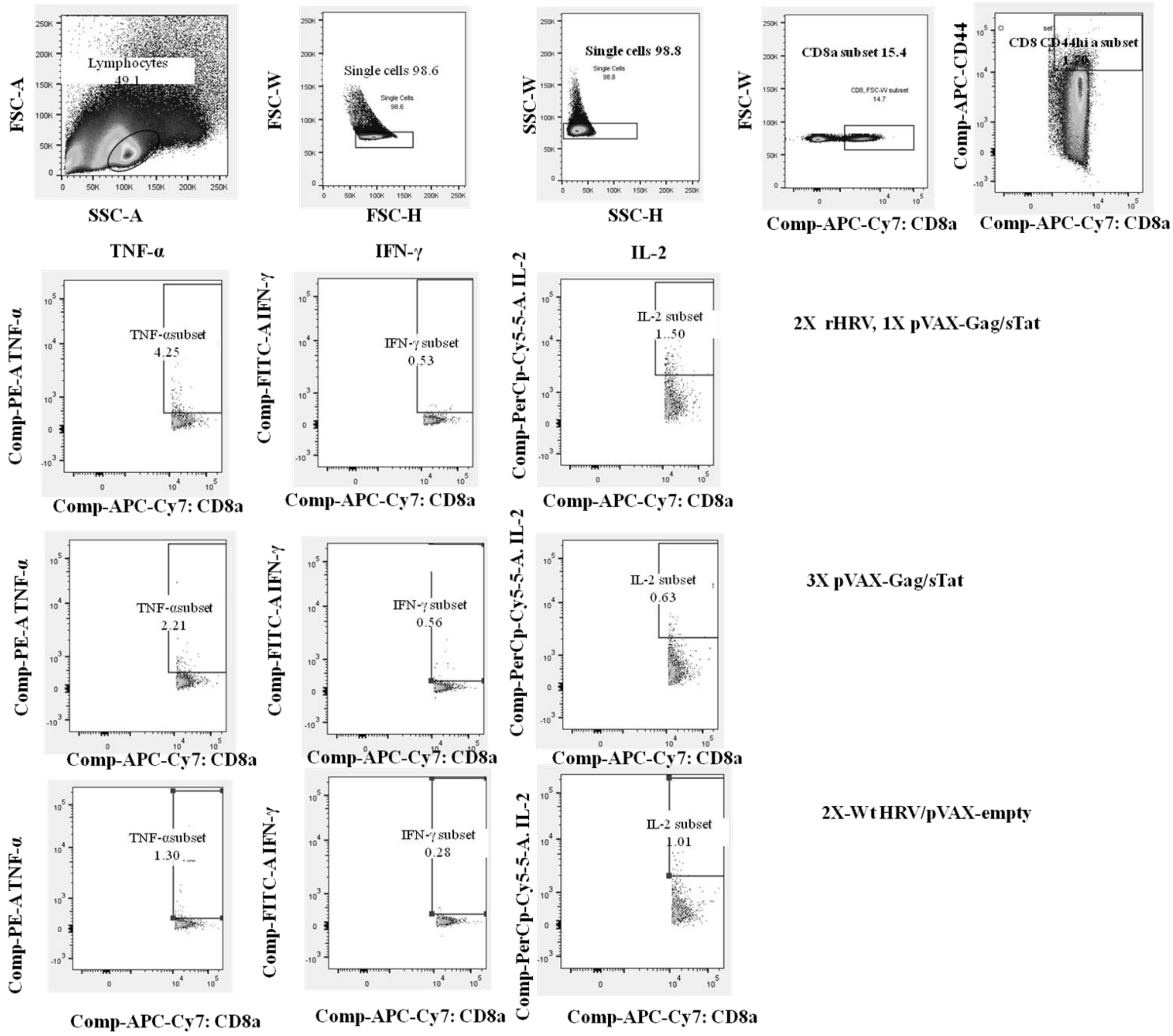

Supplementary Fig S5. Gating strategy used to detect polyfunctional effector memory CD8^{+}T cells in the spleen. Mice were vaccinated as described in the legend to figure 1and lymphocytes from mesenteric lymph nodes harvested 14 days after the final dose. Splenocytes were gated on the lymphocyte population, followed by doublet discrimination, and then gated on CD44^{hi}CD8^{+}T cells to assess the frequency of IFN-γ, TNF-α and IL-2. Representative plots for IFN-γ, TNF-α and IL-2 positive cells are shown.

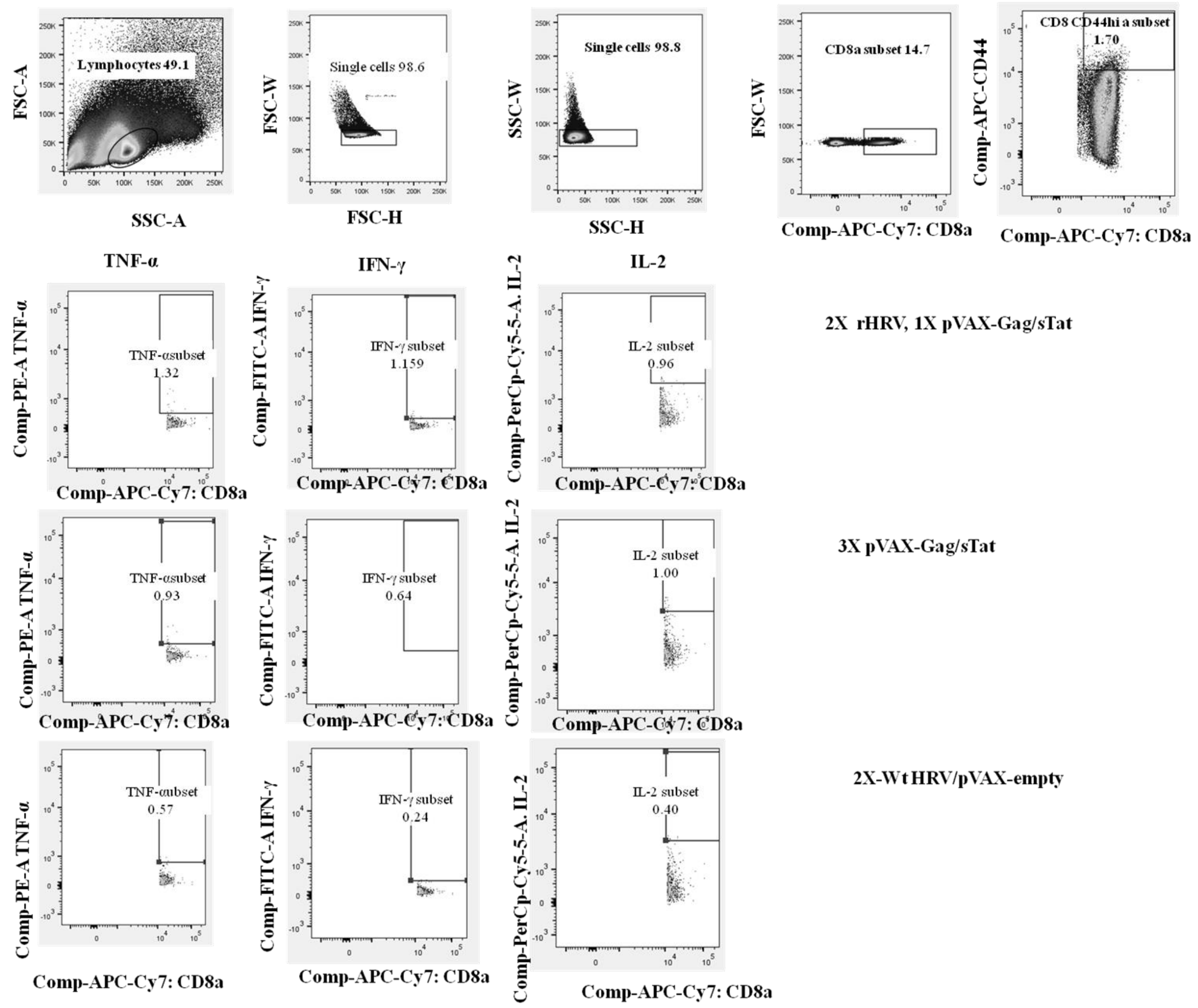

Supplementary Fig S6. Gating strategy used to detect polyfunctional effector memory $CD8^+$T cells in the spleen. Mice were vaccinated as described in the legend to figure 1and lymphocytes from mesenteric lymph nodes harvested 14 days after the final dose. Mesenteric lymphocytes were gated on the lymphocyte population, followed by doublet discrimination, and then gated on $CD44^{hi}CD8^+$T cells to assess the frequency of IFN-γ, TNF-α and IL-2. Representative plots for IFN-γ, TNF-α and IL-2 positive cells are shown.

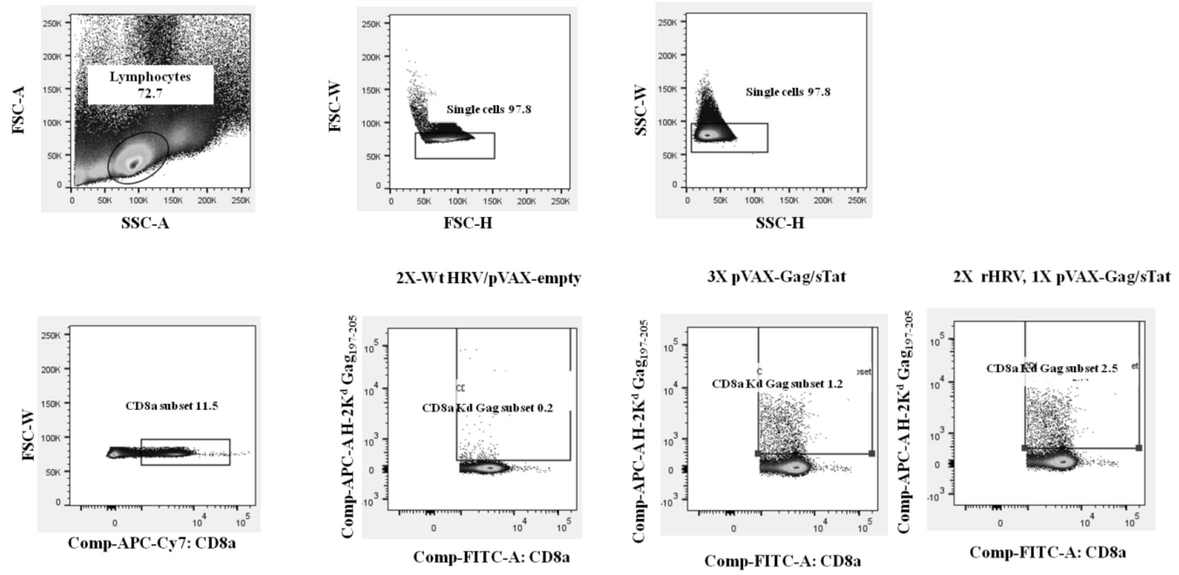

Supplementary Fig S7. Gating strategy to detect $CD8^+$ T cells in the spleen staining positive with the H-2K^d-restricted $Gag_{197\text{-}205}$ peptide. Mice were vaccinated as described in the legend to figure 1and lymphocytes from mesenteric lymph nodes harvested 14 days after the final dose. Splenocytes were gated on the lymphocyte population, followed by doublet discrimination, and then on H-2K^d-restricted $Gag_{197\text{-}205}$ $CD8^+T^+$
